# Supplementary material for: Impaired Audiovisual Representation of Phonemes in Children with Developmental Language Disorder
Source: Brain Sci. 2021 Apr 16;11(4):507. doi: 10.3390/brainsci11040507 (PMC8073635; doi:10.3390/brainsci11040507)
Supplement: Supplementary file 1 [file brainsci-11-00507-s001.zip › brainsci-1136521_supplementary.pdf]

Supplementary Materials

Table S1. Numbers of useable trials in each group for each condition and each stimulus type.

Neutral Condition						
	Standards			Deviants		
Group	Mean	Range	SE	Mean	Range	SE
DLD	195	126-221	5.7	81	40-94	3.1
TD	206	147-233	5.1	86	66-94	1.8
Audiovisual Violation Condition						
	Standards			Deviants		
Group	Mean	Range	SE	Mean	Range	SE
DLD	199	110-228	6.3	82	49-95	2.6
TD	209	152-233	4.7	87	62-94	1.8

Table S2. Models of Accuracy (72 repeated observations / 36 subjects)

	Main Effects Model					Interaction Model				
	b	*p*-value	95% CI		b_{std}	b	*p*-value	95% CI		b_{std}
fixed effects										
Group (DLD v. TD)	-2.34	0.06	-4.75	0.07	-0.60	-2.49	0.050	-4.98	0.00	-0.64
Condition (AV violation v. neutral)	-0.38	0.23	-0.99	0.24	-0.10	-0.52	0.246	-1.40	0.36	-0.13
GroupXCondition						0.29	0.648	-0.95	1.53	0.07
random effects										
intercept	12.71		7.64	21.16		12.69		7.62	21.15	
residual	1.77		1.11	2.83		1.81		1.13	2.92	

b_{std} is a partially standardized coefficient where the outcome is standardized, comparable to a conditional Cohen's d

Table S3. Models of Response Time (72 repeated observations / 36 subjects)

	Main Effects Model					Interaction Model				
	b	*p*-value	95% CI		b_{std}	b	*p*-value	95% CI		b_{std}
fixed effects										
Group (DLD v. TD)	-24.74	0.429	-86.10	36.62	-0.26	-32.00	0.310	-93.84	29.84	-0.34
Condition (AV violation v. neutral)	11.95	0.003	4.01	19.89	0.13	4.69	0.397	-6.16	15.54	0.05
GroupXCondition						14.52	0.064	-0.83	29.87	0.15
random effects										
intercept	8672.79		5347.73	14065.25		8682.39		5356.56	14073.21	
residual	295.21		184.78	471.64		275.99		171.57	443.96	

b_{std} is a partially standardized coefficient where the outcome is standardized, comparable to a conditional Cohen's d

Table S4. Models of Posterior MMN (720 repeated observations / 36 subjects)

	Main Effects Model					Best Model				
	b	*p*-value	95% CI		b_{std}	b	*p*-value	95% CI		b_{std}
*fixed effects**										
Group (DLD v. TD)	0.79	0.239	-0.52	2.10	0.26	0.04	0.955	-1.31	1.38	0.01
Condition (AV violation v. neutral)	-1.05	0.000	-1.36	-0.74	-0.35	-1.80	0.000	-2.23	-1.37	-0.61
Hemisphere (right v. midline)	-0.24	0.277	-0.66	0.19	-0.08	-0.24	0.269	-0.65	0.18	-0.08
Hemisphere (left v. midline)	0.22	0.312	-0.21	0.64	0.07	0.22	0.304	-0.20	0.64	0.07
Hemisphere (right v. left)	-0.45	0.010	-0.80	-0.11	-0.15	-0.45	0.009	-0.80	-0.11	-0.15
GroupXCondition						1.49	0.000	0.88	2.10	0.50
random effects										
intercept	3.79		2.29	6.27		3.80		2.30	6.28	
residual	4.50		4.04	5.01		4.36		3.91	4.85	

b_{std} is a partially standardized coefficient where the outcome is standardized, comparable to a conditional Cohen's d
*models control for electrode site

Table S5. Models of Anterior MMN (720 repeated observations / 36 subjects)

	Main Effects Model					Best Model				
	b	*p*-value	95% CI		b_{std}	b	*p*-value	95% CI		b_{std}
*fixed effects**										
Group (DLD v. TD)	0.46	0.274	-0.36	1.28	0.20	0.00	0.995	-0.86	0.87	0.00
Condition (AV violation v. neutral)	-0.37	0.008	-0.64	-0.10	-0.16	-0.82	0.000	-1.20	-0.44	-0.37
Hemisphere (right v. midline)	-0.83	0.000	-1.20	-0.45	-0.37	-0.83	0.000	-1.19	-0.46	-0.37
Hemisphere (left v. midline)	-0.46	0.014	-0.84	-0.09	-0.21	-0.46	0.013	-0.83	-0.10	-0.21
Hemisphere (right v. left)	-0.36	0.019	-0.66	-0.06	-0.16	-0.36	0.019	-0.66	-0.06	-0.16
GroupXCondition						0.91	0.001	0.38	1.45	0.41
random effects										
intercept	1.42		0.83	2.42		1.42		0.84	2.42	
residual	3.44		3.09	3.83		3.39		3.04	3.77	

b_{std} is a partially standardized coefficient where the outcome is standardized, comparable to a conditional Cohen's d
*models control for electrode site

Table S6. Models of P3 (576 repeated observations / 36 subjects)

	Main Effects & Best Model				
	b	*p*-value	95% CI		b_{std}
*fixed effects**					
Group (DLD v. TD)	-0.30	0.681	-1.76	1.15	-0.10
Condition (AV violation v. neutral)	-0.20	0.267	-0.54	0.15	-0.06
Hemisphere (right v. midline)	-1.15	0.000	-1.61	-0.69	-0.38
Hemisphere (left v. midline)	-0.31	0.189	-0.76	0.15	-0.10
Hemisphere (right v. left)	-0.84	0.000	-1.27	-0.42	-0.28
random effects					
intercept	4.66		2.81	7.71	
residual	4.49		3.98	5.07	

b_{std} is a partially standardized coefficient where the outcome is standardized, comparable to a conditional Cohen's d
*models control for electrode site
